# Effectiveness and safety of fire needle for knee osteoarthritis: A protocol of systematic review and meta-analysis

**DOI:** 10.1097/MD.0000000000023962

**Published:** 2021-01-22

**Authors:** Lunbin Lu, Jing Ye, Jun Xiong, Jun Chen, Siyuan Zhu, Zhiying Zhong, Genhua Tang, Xingchen Zhou, Han Guo

**Affiliations:** a Jiangxi University of Traditional Chinese Medicine; b The Affiliated Hospital of Jiangxi University of Traditional Chinese Medicine, Nanchang, China.

**Keywords:** efficacy evaluation, fire needle, knee osteoarthritis, meta-analysis, protocols, randomized controlled trials

## Abstract

**Background:**

There is a lack of curative medical treatment for patients with knee osteoarthritis (KOA). Acupuncture represents an important alternative therapy. In various forms of acupuncture and moxibustion, the fire needle is an indispensable part. Knee osteoarthritis (KOA) is a series of symptoms and signs of knee joint caused by local injury and inflammation and chronic strain of the knee joint resulting in cartilage degeneration of the articular surface and reactive bone loss of the subchondral bone plate. The results of clinical trial indicated that the fire needle therapy has obvious curative effect in treating KOA. This protocol is intended to describe how to collate and accumulate evidence for the current efficient and safe treatment of KOA with fire needle.

**Methods:**

Seven electronic databases were used to retrieve the literature for the KOA randomized controlled trials, including 3 English databases (PubMed, EMBASE, the Cochrane Central Register of Controlled Trials [Cochrane Library]), and 4 Chinese databases (Chinese National Knowledge Infrastructure, Chinese VIP Information, Wanfang Database, and Chinese Biomedical Literature Database). This systematic review will include all randomized controlled clinical trials using fire needle therapy for KOA. The observation Index is the Change of Western Ontario and McMaster Universities Osteoarthritis Index Total, first proposed by Bellamy in 1988. The selection of the study will be completed independently by 2 reviewers, extract the data, and evaluate the quality of the study before selecting the title, abstract, and full text. Revman 5.4 software will be used to perform meta-analyses of randomized controlled trials, where risk ratios for dichotomous data and standardized or weighted mean differences for continuous data are the results.

**Result:**

The results will be published in a peer-reviewed journal.

**Conclusion:**

This systematic review will provide the latest evidence to evaluate the safety and efficacy of fire needle therapy in patients with KOA.

**Trial registration number:**

INPLASY202080030

## 1 Introduction

### 1.1 Description of the condition

Osteoarthritis (OA)^[1]^ is a common orthopedic disease due to the degenerative changes of synovial articular cartilage and the chronic joint disease characterized by narrowing of joint space, formation of articular marginal osteophytes, and subchondral osteosclerosis. It is mostly found in joints with large weight bearing, especially knee joints. According to literature reports,^[2]^ it accounts for about 31% of all osteoarthritis of the whole body. The lesions are characterized by degenerative changes of articular cartilage and secondary hyperosteogeny at the joint margin. Joint pain, tenderness, sound, joint effusion as the main symptoms of the disease. When the disease gradually worsens, the patient's joint activity is limited and joint deformity occurs over time. Moreover, knee osteoarthritis (KOA) is difficult to cure and the disease is prone to relapse, which affects the patient's work and life to different degrees. In severe cases, the patient cannot take care of himself. To bring serious social burden to the family and the society.^[3]^ The incidence of KOA is estimated to be close to 0.02% in the U.S. population, often leading to early retirement and joint replacement.^[4]^ The overall KOA prevalence in Spain, Norway, Greece, and Italy was 12.2%, 7.1%, 6.0%, and 5.4%, respectively.^[5]^ Modern medicine believes that its etiology and pathogenesis are very complex, not the result of a single factor, and related to the biomechanical balance of joint cartilage degeneration, osteoporosis, and so on. In terms of treatment, there is still a lack of recognized specific drugs, and most of them focus on improving symptoms, delaying progression, and reducing disability.^[3]^ However, as the patients with long disease course of KOA are older, long-term use of drugs has certain toxic and side effects, and it is difficult to achieve the purpose of cure, the surgical treatment is of high risk and high cost, and the prognosis is not certain, nor can it be fundamentally cured.^[6–8]^ Many studies and meta-analyses report that intra-articular hyaluronic acid injections are widely used in the treatment of arthritis; injections of hydroxyapatite are effective because the viscoinduction properties of hydroxyapatite increase joint lubrication.^[9]^ However, intra - joint injections, which usually relieve pain and enhance joint function, do not work in severe KOA patients.^[10]^ Lacking curative medical treatment for KOA, 4current management follows a stepwise approach that begins with conservative treatment with medications such as non-steroidal anti-inflammatory drugs, glucosamine, topical analgesics, intra-articular injections (corticosteroids, hyaluronic acid, blood-derived products), and ends with arthroscopic knee surgery (largely discredited in randomized trials) and knee replacement.^[6]^ The age-standardised incidence rates for knee replacement surgery were estimated at 150 per 100000 person years in Western countries.^[11]^

### 1.2 Description of the intervention

Fire needle therapy is a treatment method in which a thick and thin needle made of a special material is burned red on the fire and then quickly penetrated into certain points and parts of the human body. The fire needle combines the double function of acupuncture and moxibustion, with the heat of fire and the dredge function of acupuncture, it exerts the effect of activating blood and relieving pain.^[12]^ It has good curative effect on KOA with long course and deep position.^[13]^ Modern clinical reports indicate that the fire needle can destroy the diseased tissues around the knee joint by its warm stimulation, stimulate autoimmunity, and produce antibodies, and accelerate the absorption of necrotic tissues.^[14]^ Not only that, the fire needle will have a good effect on the nerve center, so that the pain excitement center is inhibited, reduce pain, so that the disease turned over.^[13]^

Fire needle therapy has a significant effect on KOA, which is easy to use, safe and reliable, and has no toxic side effects. The purpose of this review is to summarize clinical studies on the treatment of KOA with fire needle, and the results of this review will be reliable in the presence of clinical research evidence. This article reviews the effects of fire needle on KOA and does not discuss other effective treatments.

### 1.3 The reason to perform this overview

The purpose of this study was to systematically review the existing literature to assess the efficacy and safety of fire needle therapy for KOA.

## 2 Methods

### 2.1 Registration

The protocol has been registered on the International Platform of Registered Systematic Review and Meta-analysis Protocols (INPLASY) (registration number, INPLASY202080030; https://inplasy.com/inplasy-2020-8-0030/) basing on the Preferred Reporting Items for Systematic Reviews and Meta-Analyses Protocols (PRISMA-P) statement guidelines.^[15]^

### 2.2 Inclusion criteria for study selection

#### 2.2.1 Types of studies

All available randomized controlled trials (RCTs) on fire needle treatment for KOA will be included. Others such as retrospective study, case report, review, and studies which use inappropriate random sequence generation methods will be excluded. Language will be restricted to Chinese and English.

#### 2.2.2 Participants

Participants aged 40 years or older and diagnosed with mild or moderate KOA are eligible to participate in the study. The following criteria are used for diagnosis of KOA according to the Chinese Guideline for the Medical Management of Osteoarthritis^[16]^:

(1) refractory knee pain for most days in the last month;
(2) joint space narrowing, sclerosis or cystic change in subchondral bone (as demonstrated by X-ray);
(3) laboratory examinations of arthritis: clear and viscous synovial fluid (≥2 times) and white cell count <2 × 10̂9/L;
(4) aged 40 years or older;
(5) morning stiffness continues less than 30 minutes;
(6) bone sound exists when joints was taking flexion and/or extension. If a patient meets the following combination of criteria (1 and 2), (1, 3, 5 and 6), or (1, 4, 5 and 6), a diagnosis of KOA is confirmed.

#### 2.2.3 Interventions

The purpose of the study is on clinical trials of fire needle treatment for KOA.^[17]^ Studies applied fire needle in the experimental group will be included. Fire needle combined with other therapies will be excluded if the efficacy of fire needle cannot be clarified in the combined therapy. The therapeutic intervention of controlled group can be conventional acupuncture, electro-acupuncture, auriculo-acupuncture, or pharmcological therapy.

#### 2.2.4 Outcomes

The primary outcome is the change of Western Ontario and McMaster Universities Osteoarthritis Index (WOMAC)^[18]^ total score from baseline to 16 weeks. The WOMAC uses 24 parameters to evaluate patients with knee osteoarthritis, and is also used to monitor the progress of the disease. It is an internationally recognized evaluation standard and widely used in clinical practice. It has been translated and validated in different languages including Chinese.^[19]^ The Chinese-language version of WOMAC has 24 items to evaluate pain (5 items), stiffness (2 items), and physical function (17 items) in KOA patients. Each of the 24 items will be scored using a visual analogue scale,^[20]^ on a scale of 0 to 10, with a higher score indicating greater pain, stiffness, and poor physical functioning. The pain, stiffness, and physiological function subscales range from 0 50, 0 20, and 0 170, respectively, for a total score of 0 240.

### 2.3 Search strategy

#### 2.3.1 We will search the following databases:

1. The Cochrane Skin Group Trials Register (the inception to 2020.10);
2. MEDLINE (the inception to 2020.10);
3. EMBASE (the inception to 2020.10);
4. The Cochrane Central Register of Controlled Trials (CENTRAL; the inception to 2020.10);
5. Chinese Biomedical Literature Database (CBM; the inception to 2020.10);
6. Chinese Medical Current Content (CMCC; the inception to 2020.10);
7. China National Knowledge Infrastructure (CNKI; the inception to 2020.10).

This review will use the following search terms: knee osteoarthritis, KOA, acupuncture, electro-acupuncture, auricular acupuncture, elongated needle, fire needling, moxibustion. This study will adapt this strategy to search all the above databases. There will be no restriction on language or publication type. The search strategy for MEDLINE can be found in online supplementary appendix 1.2.4.2. Searching other resources. Relevant systematic review or meta-analysis of RCTs will be electronically searched. Moreover, we will filter relevant medical journals and magazines to identify literature which is not included in the electronic databases. The preliminary search strategy for PubMed is presented in Table 1.

**Table 1:** The search strategy for PubMed.

Order	Strategy
#1	Search “knee osteoarthritis” [MeSH] sort by: publication date
#2	Search ((knee osteoarthritis[title/abstract]) or koa [title/abstract]) sort by: publication date
#3	#1 or #2
#4	Search (((((((randomized controlled trial [publication type]) or controlled clinical trial [publication type]) or randomized [title/abstract]) or drug therapy [MeSH subheading]) or placebo [title/abstract]) or randomly [title/abstract]) or trial [title/abstract]) or groups [title/abstract] sort by: publication date
#5	Search (humans [MeSH terms]) not animals [MeSH Terms] sort by: publication date
#6	#4 and #5
#7	Search “acupuncture” [MeSH] sort by: publication date
#8	Search (((Acupuncture [Title/Abstract]) or fire needle [title/abstract]) or fire needling [title/abstract]) sort by: publication date
#9	#7 or #8
#10	#3 and #6 and #9

### 2.4 Study selection

**Figure 1:**
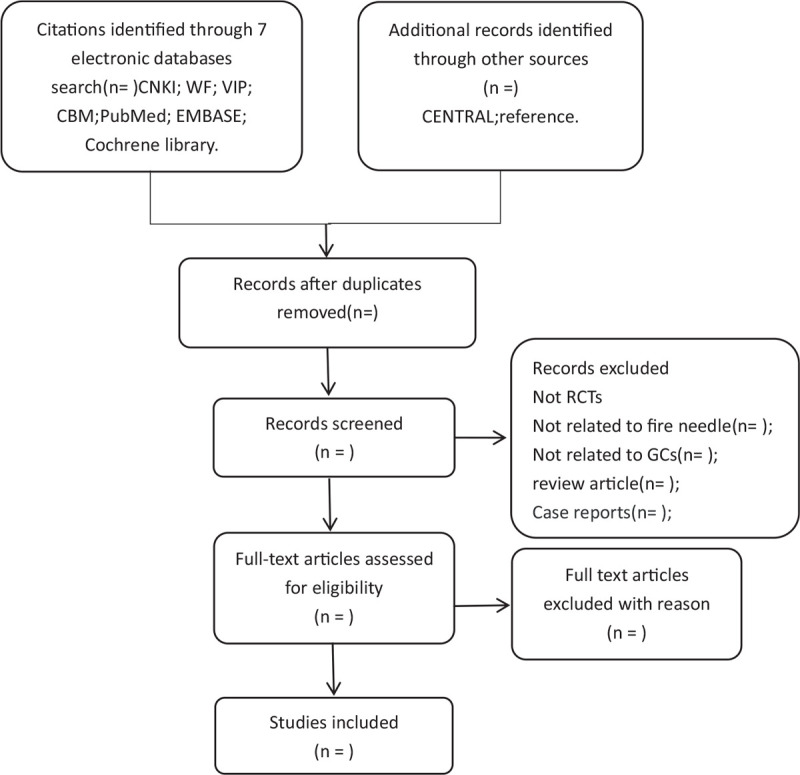
Flowchart of literature selection.

We will select the RCTs comparing the effect and safety of fire needle therapy on KOA. Those articles meeting 1 of following items will be excluded:

1. the duplicates,
2. the participants did not meet the diagnosis criteria of KOA or the diagnosis criteria is unknown,
3. not RCT studies,
4. the studies in which the experimental participants do not receive fire needle therapy in combination with conventional therapy as the primary intervention,
5. the intervention contains any other traditional Chinese medicine therapy,
6. incomplete data which will be needed. The studies whether are eligible will be assessed by 2 authors. When there is any disagreement during the articles inclusion, we will discuss to solve it. The specific process of studies screening will be displayed in a PRISMA flow diagram (Fig. 1).

### 2.5 Data extraction and management

To ensure that necessary information would not be lost, each of the SRs and Meta-analyses will be submitted to data extraction by 2 independent reviewers (LBL and JC) the following data will be extracted:

(1) Study characteristics: author, country, year of publication, study design, and database;
(2) Population characteristics: sex, age, baseline diseases, and sample size;
(3) Methodological characteristics: information sources, intervention(s), comparison(s), bias assessment, and funding, etc.

If there are any differences, we will resolve them by mutual discussion or by consensus with the third reviewer (JX).

### 2.6 Data analysis

#### 2.6.1 Assessment of risk of bias (ROB) in included studies

Two authors (SYZ and LBL) evaluated the quality and ROB of the included SR/Meta-analysis independently using the ROB in Systematic reviews (ROBIS) tool and PRISMA.^[21]^ A consensus is reached by 2 reviewers through discussion, and independent decisions are made by experts (JX) if necessary.

#### 2.6.2 Measures of treatment effect

For continuous variables, we will use mean difference to evaluate the extracted data. For dichotomous variables, rate ratio will be applied to analyze. The confidence intervals for both continuous and dichotomous variables will be set to 95%.

#### 2.6.3 Dealing with missing data

If the specific information is not be reported, we will attempt to contact the original author for relevant information. If the required information is not available, it will be explained in the article. Then, the analysis will rely on existing data, and discuss the potential impact of missing information.

#### 2.6.4 Assessment of heterogeneity

The heterogeneity will be evaluated with the use of I^2^ values in accordance with the Cochrane Handbook (0%–40%, might not be important, 30%–60%, may represent moderate heterogeneity, 50%–90%, may represent substantial heterogeneity, and 75%–100% may represent considerable heterogeneity). We will select the random effects model and then further subgroup analysis will be performed to investigate the possible causes of heterogeneity, if the heterogeneity among trials is significant (I^2^ ≥ 50%). Conversely, we will choose the fixed effect model, if an I^2^ values less than 50%.

#### 2.6.5 Assessment of reporting bias

Funnel plot will be used to assess reporting biases of the studies include. We will consider that the reporting bias is existing and the reliability is low if the points on both sides of the funnel plot are dispersed and asymmetrical. Conversely, if the points on either side of the funnel plot are symmetrically distributed in substantial, we will consider the reporting bias as non-existent and the result is reliable.

#### 2.6.6 Data synthesis and subgroup analysis

We will use RevMan software (V5.4, The Nordic Cochrane Centre, The Cochrane Collaboration, Copenhagen, Denmark) to conducted all analyses. And we will select a random effects model or fixed effects model to merge the primary and secondary outcome indicators in accordance with the results of heterogeneity test. We will apply the fixed effects model for data synthesis of low heterogeneity (I^2^<50%) while the random effects model will be conducted if the heterogeneity is significant (I^2^ ≥ 50%). It is considered that differences are statistically significant if the results of Z test show that *P* value is less than .05, and the 95% CI does not contain 0 (for continuous variables) or the 95% CI does not contain 1 (for dichotomous variables). If heterogeneity is evaluated as significant (I^2^ ≥ 50%) and the trials included are adequate, we will perform a subgroup analysis to explore the potential source of the heterogeneity according to the difference in participant characteristics, interventions, controls, and outcome measures.

#### 2.6.7 Sensitivity analysis

We will carry out sensitivity analysis to identify the quality and robustness of the meta analysis result when the outcome analyses involve a large degree of heterogeneity, according to sample size, methodological quality, and the effect of missing data.

#### 2.6.8 Grading the quality of evidence

We will evaluate the quality of evidence and rate it into 4 levels: high, moderate, low, or very low in accordance with the Recommendations Assessment, Development and Evaluation guide lines.^[22]^

#### 2.6.9 Ethics and dissemination

Ethical approval will not be necessary because the data included in our study are derived from published literature and are not linked to individual patient data. The systematic review providing implication of the effectiveness and safety of fire needle for will be published in a peer reviewed journal or conference presentations.

## 3 Discussion

KOA is a major health problem worldwide and its disease burden continues to increase as the population ages. Many current standard treatments for KOA, such as nonsteroidal anti-inflammatory drugs, intra-articular injections, and glucosamine, are often ineffective. Because of the limitations of these alternative therapies, the fireneedle has become a long-term alternative therapy in China and is receiving increasing attention in many Western countries.^[23]^ Some RCT has been performed to test the effectiveness of fire against KOA. Based on published systematic reviews and meta-analyses of these RCTS, Fire Needle appears to be a promising approach for KOA treatment.^[24]^

Acupuncture and moxibustion, as a main non-drug therapy, is widely used in the treatment of arthritis. Studies have shown that acupuncture can effectively relieve pain and improve the mobility of knee joints.^[25]^ Fire needle is a combination of acupuncture and heat. It has good curative effect on KOA by warming meridian and collaterals.^[12]^ Studies have indicated that fire acupuncture can not only increase local temperature, accelerate blood circulation and improve metabolism, promote the exudation of white blood cells and increase their phagocytic function, promote the disappearance of inflammation, and nourish local skin tissues.^[26–27]^

The evaluation of this literature shows that the operation of fire needle in the treatment of KOA is safe and effective, and the cure rate and effective rate of fire needle therapy.^[23]^ However, this study may have limitations that might limit its ability to generate conclusions based on high confidence. Specifically, there may be significant heterogeneity in the forms of fire needle used and the qualities of methodology. There will also most likely be differences in outcomes measured and tools used. Inherent uncertainty exists by pooling these data within constructed domains. More electronic databases or Gray studies were not searched in this systematic review, which may affect the retrieval and results of RCTs. This review is registered on INPLASY (registration number, INPLASY202080030, https://inplasy.com/inplasy-2020-8-0030/)

## Author contributions

All authors have read and approved the publication of the protocol.

**Conceptualization:** Lunbin Lu.

**Data curation:** Lunbin Lu, Jing Ye, Jun Xiong.

**Formal analysis:** Lunbin Lu.

**Methodology:** Jing Ye, Jun Chen, Siyuan Zhu, Zhiying Zhong, Genhua Tang, Xingchen Zhou, Han Guo.

**Resources:** Jun Chen, Siyuan Zhu, Zhiying Zhong, Genhua Tang, Xingchen Zhou, Han Guo.

**Supervision:** Jun Xiong.

**Writing – original draft:** Lunbin Lu.

**Writing – review & editing:** Jun Xiong.
